# A service evaluation of the healthy lifestyle groups in a female medium secure unit- what do our patients know about nutrition?

**DOI:** 10.1192/bjo.2021.917

**Published:** 2021-06-18

**Authors:** Lauren Shipperbottom, Ruth Scally

**Affiliations:** 1 University of Birmingham, Medical School; 2 Ardenleigh

## Abstract

**Aims:**

To assess whether patients have a good knowledge of basic nutrition compared to a group of staff. We hypothesise that the patient's knowledge will show deficits compared to the staff despite the group interventions.

**Background:**

The Royal College of Psychiatrist's core standards for inpatient physical health outlines that patients should be engaged in healthy lifestyle groups. The women's secure service at Ardenleigh has developed healthy lifestyles groups to promote a better understanding of nutrition.

**Method:**

An adapted University College London general knowledge nutrition questionnaire was used to investigate nutritional knowledge.

All 22 inpatients and a random selection of staff were offered the chance to complete the questionnaire. As the groups run on a regular basis, it was presumed all patients had attended at least one group session. The staff are the comparator group.

18 staff responses and 12 inpatient responses were obtained (54.5% response rate for inpatients).

**Result:**

No participant in either group scored 100%. Both groups had a good awareness of what foods they should be eating more and less of. 83.3% of patients were aware that they should be eating breakfast everyday as opposed to 100% of staff.

Poor areas of knowledge included knowledge of the number of oily fish servings per week. Staff and patients also performed poorly when estimating their recommended daily salt intake. 1/3 of patients were unable to provide an example of a serving of fruit and vegetables.

The knowledge of the structure of the Eat-Well plate was poor in both groups. Only 16% of patients and 22% of staff were aware that starchy foods should make up 1/3 of the Eat-well plate. Knowledge of protein sources was poor. 25% of patients and 16.6% of staff thought that fruit and butter were good sources of protein

Furthermore, only 50% of patients were able to choose the healthiest evening meal choice from a list of 3 options compared to 100% of staff.

**Conclusion:**

In conclusion staff had better knowledge of nutrition than patients but knowledge was poor in areas amongst both groups. We conclude that groups should have more focus around practical applications of nutritional knowledge to everyday life.

